# A novel forkhead box C1 gene mutation in a Korean family with Axenfeld-Rieger syndrome

**Published:** 2013-04-30

**Authors:** Gyu-Nam Kim, Chang-Seok Ki, Seong-Wook Seo, Ji-Myong Yoo, Yong-Seop Han, In-Young Chung, Jong-Moon Park, Seong-Jae Kim

**Affiliations:** 1 Department of Ophthalmology, Gyeongsang National University, College of Medicine, Jinju, Korea; 2 Gyeongsang Institute of Health Science, Gyeongsang National University, Jinju, Korea; 3 Department of Laboratory Medicine and Genetics, Samsung Medical Center, Sungkyunkwan University School of Medicine, Seoul, Korea

## Abstract

**Purpose:**

To report a case series of patients with novel forkhead box CI (*FOXC1*) mutations in a Korean family with Axenfeld-Rieger syndrome (ARS).

**Methods:**

Four members of the same family underwent complete ophthalmologic and systemic examinations and genetic analysis. Genomic DNA was isolated from peripheral blood leukocytes, and all coding exons with flanking intronic regions of the *FOXC1* and pituitary homeobox 2 genes were amplified using PCR, and sequenced.

**Results:**

The patients were 40, 12, 11, and 10 years old (father, son, and two sisters, respectively). All four had uncontrolled intraocular pressure, glaucomatous visual field defect, retinal nerve fiber layer defect, iridocorneal adhesion on gonioscopy, hypoplasia and marked atrophy of the iris, flattening of the midface, and broad flat noses. A diagnosis of ARS was made based on characteristic ocular and systemic traits. A novel *FOXC* mutation, c.317delA, was identified in all affected members of the family with ARS.

**Conclusions:**

We found a novel c.317delA mutation in *FOXC1* in a Korean family with ARS. We suggest that this *FOXC1* mutation causes typical ARS, and that our results may be useful for better understanding of the spectrum of *FOXC1* mutations and the role of FOXC1 in the development and progression of ARS.

## Introduction

Axenfeld-Rieger syndrome (ARS) is characterized by anomalies of the anterior segment of the eye and systemic signs, and is inherited in an autosomal dominant pattern [1]. The anterior segment anomalies include iris strands connecting the iridocorneal angle to the trabecular meshwork and a prominent, anteriorly displaced Schwalbe’s line (posterior embryotoxon), iris hypoplasia, abnormal situation of pupil (corectopia), more than one pupil in an eye (polycoria), and glaucoma [2,3]. The systemic signs include abnormality of the cardiovascular outflow tract, midface hypoplasia, a broad flat nasal root, maxillary and occasionally mandibular hypoplasia, hypertelorism (excessive distance between two eyes) and telecanthus (abnormally increased distance between the medial canthi of the eyelids), microdontia (abnormally small teeth), hypodontia (having fewer teeth than normal), skeletal anomalies, and hearing loss. The phenotype of this syndrome varies considerably among cases and even in the two eyes of the same individual [2,4,5].

ARS is genetically heterogenous, and two major genes, forkhead box C1 (*FOXC1*) on chromosome 6p25 and pituitary homeobox 2 (*PITX2*) on chromosome 4q25, have been demonstrated to cause the disease [6,7]. The *FOXC1* mutation is more likely to be associated with glaucoma, while the risk of systemic abnormalities is greatest with the *PITX2* mutation. Other implicated loci include those at 13q14 and 16q24, but no definitively causative mutations have been identified in any one gene. A recent, single autosomal recessive case of a child with characteristic anterior chamber anomalies and umbilical hernia was identified as a compound heterozygote for mutations in *CYP1B1* [8-10].

The *FOXC1* gene is a member of the forkhead family of transcription factors, which play important roles in embryogenesis, tissue-specific gene expression, and tumor development. *FOXC1* recognizes and binds to specific DNA sequences through the conserved 110-amino-acid forkhead domain (FH), and thus activates the target genes [11]. The most common *FOXC1* defects leading to ARS are point mutations. More than 40 documented deletions, either interstitial or telomeric, involve 6p25, and patients frequently present with ocular, craniofacial, skeletal, and cardiac malformations and hearing loss. The phenotypic variation seen in these patients is largely due to the size of the deletions and the genes involved [12-14]. To the best of our knowledge, intragenic mutations, including deletions, of *FOXC1* have been described in 46 patients, but c.317delA has not been detected in any patients with ARS, including those with ocular and systemic abnormalities [15]. Here, we describe the c.317delA genetic aberration in a Korean family and the clinical phenotypes of ARS in these patients.

## Methods

**Figure 1:**
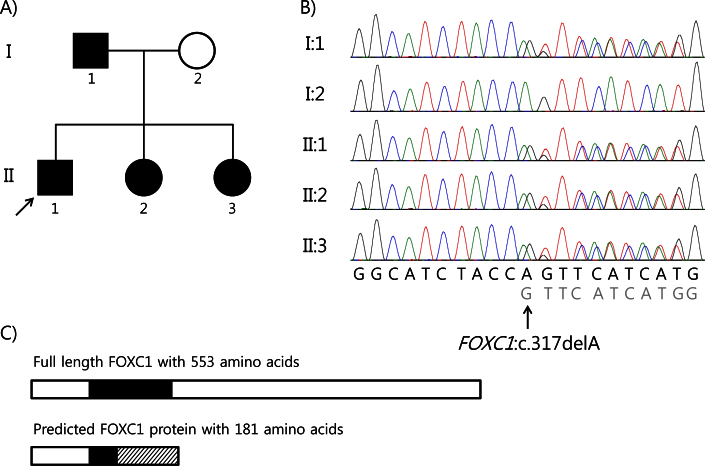
Pedigree and sequence analysis of the forkhead box CI (*FOXC1*) gene in a family with Axenfeld-Rieger syndrome. **A**: The proband (II:1) is indicated with an arrow. The filled symbols represent affected individuals. **B**: Direct sequencing analysis of the *FOXC1* gene showed overlapping peaks at nucleotide position 317 due to a heterozygous 1-bp deletion of alanine (c.317delA) in the proband (II:1), his father (I:1), and two younger sisters (II:2 and II:3). **C**: The FOXC1 domain structure and predicted protein due to a novel mutation (c.317delA; p.Gln106Argfs*75) identified in this study are illustrated. The black rectangle represents the forkhead domain, and the slashed box indicates those areas of the mutant protein that are translated differently from the normal FOXC1 protein.

The purpose of the study and the procedures were explained to all patients, and informed consent was obtained. The procedures used conformed to the tenets of the Declaration of Helsinki. This study was approved by the Gyeongsang National University Institutional Review Board. The proband (Figure 1A) in this Korean family was a 12-year-old boy who presented with decreased visual acuity and intraocular pressure (IOP) uncontrolled by antiglaucoma medications in the right eye. His best-corrected vision was 20/200 in the right eye and 20/20 in the left eye. The IOP measured with Goldmann applanation tonometry was 36 mmHg in the right eye and 24 mmHg in the left. Ocular examination revealed normal corneal diameter, Haab’s striae (horizontal break in Descemet’s membrane), and iris atrophy in both eyes. In addition, gonioscopy showed open angles in both eyes with anterior insertion of the iris into the trabecular meshwork with prominent iris processes. He had apparent hypertelorism, telecanthus, a flat face, and a flat, broad nasal bridge. Because of these findings, he was diagnosed with bilateral ARS.

This family had four affected members: the proband, his father, and his two younger sisters (Figure 1A). Table 1 summarizes the clinical characteristics of the ocular and systemic examination of the four patients in this family.

**Table 1:** Clinical features of the family.

Parameters	Patient I:1		Patient II:1		Patient II:2		Patient II:3	
	OD	OS	OD	OS	OD	OS	OD	OS
Ocular characteristics								
BCVA	0.5	LP(-)	0.1	1	3m FC	1	1	1
Refraction(spherical equivalent)	plano		+0.50sph	+0.50sph	+2.00sph	−1.50sph	−0.75sph	+0.25sph
IOP (mmHg)	26	36	36	24	32	24	24	30
Corneal diameter(mm)	12	12	12.5	12.5	12	12	11.5	11.5
ECC count(cell/mm2)	768	903	1078	1494	1323	2385	2486	1478
Central corneal thickness(µm)	529	532	541	525	573	537	584	606
Posterior embryotoxon	-	-	-	-	+(N, T)	+(N)	+(N, T)	+(N, T)
Haab’s striae	+	+	+	+	+	-	-	+
C/D ratio	Nearly total cupping	Nearly total cupping	0.9	0.6	0.5	0.3	0.5	0.5
V/F: mean deviation (db)	−32.8	−33.06	−29.1	−4.02	−8.27	−4.02	−3.61	−4.92
Systemic abnormalities								
Flat nose	+		+		+		+	
Hypertelorism	+		+		+		+	
Telecanthus	+		+		+		+	

BCVA, Best corrected visual acuity; LP, light perception; FC, finger count; ECC, endothelial cell count; N, nasal side, T, temporal side; +, present; -, not present; C/D, cup disc ratio.

### Clinical evaluation

All four patients underwent comprehensive ophthalmologic examinations, including best-corrected visual acuity measurement and slit lamp biomicroscopy, followed by ophthalmoscopy after pupillary dilation. Additional examinations included anterior segment photography, gonioscopic photography, optical coherence tomography (Carl Zeiss Meditec, Dublin, CA), visual field testing (Carl Zeiss Meditec), dental panoramic radiography, trans-thoracic echocardiography, auditory function testing, and computed tomography of the head. In addition, we obtained information about the patients’ systemic abnormalities through the clinical interview.

### Mutation analysis of the pituitary homeobox 2 and forkhead box C1 genes

Genomic DNA was isolated from peripheral blood leukocytes using the Wizard Genomic DNA Purification Kit (Promega, Madison, WI). All coding exons, with flanking intronic regions, of the *FOXC1* and *PITX2* genes were amplified using PCR with primers designed by the authors (available upon request) and a thermal cycler (Model 9700; Applied Biosystems, Foster City, CA). Sequencing was performed with the ABI Prism 3100×l Genetic Analyzer (Applied Biosystems) using the BigDye Terminator Cycle Sequencing Ready Reaction Kit (Applied Biosystems). The obtained sequences were analyzed using Sequencher software (version 4.10.1, Gene Codes, Ann Arbor, MI) and compared with the reference sequence for *FOXC1* (NM_001453.2) and *PITX2* (NM_000325.5). We followed the Human Genome Variation Society guidelines to describe sequence variations: “A” of the ATG translation start site was numbered +1 for the DNA sequence, and the first methionine was numbered +1 for the protein sequence.

## Results

### Molecular genetic analysis of the forkhead box C1 and pituitary homeobox 2 genes

**Figure 2:**
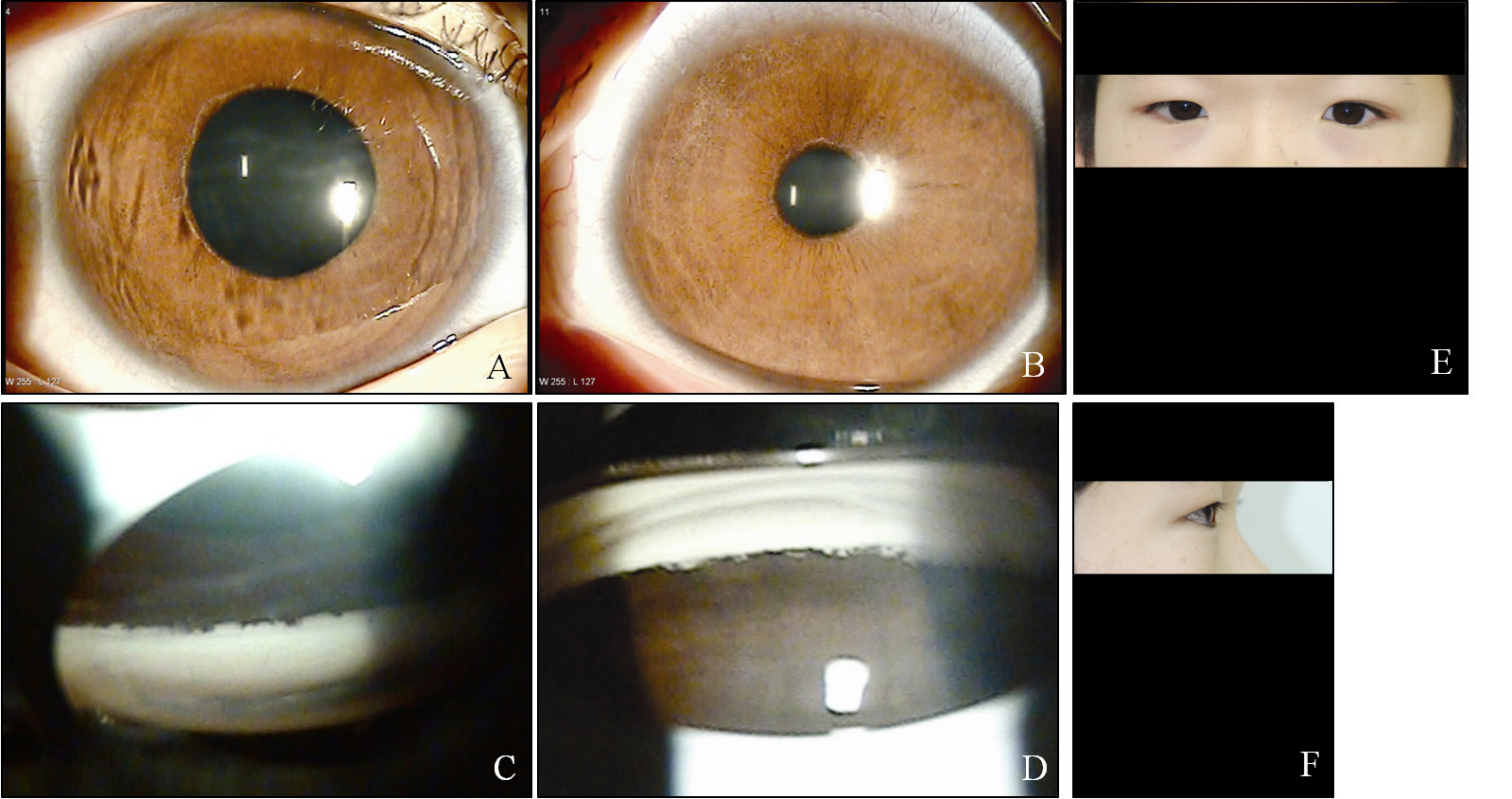
Ocular characteristics and systemic anomalies of patient II:1. Slit lamp photographs showed Haab’s striae and iris hypoplasia in both eyes (**A**, **B**). Gonioscopy showed open angles in both eyes with anterior insertion of the iris into the trabecular meshwork, prominent iris processes, and broad-based synechiae at places in both eyes (**C**, **D**). Physical examination revealed hypertelorism, telecanthus, a flat face, and flat broad nasal bridge (**E**, **F**).

The proband presented with complex bilateral anterior segment developmental anomalies including the presence of Haab’s striae (see “Patient Details”; Figure 2). The father and sisters of the proband showed a typical ARS phenotype, but the proband’s mother appeared normal, with no sign of the disease. We screened the four members of the family for coding mutations in the *FOXC1* and *PITX2* genes. The proband was heterozygous for a 1-bp deletion in the *FOXC1* gene (c.317delA) that is expected to result in ARS through haploinsufficiency either due to nonsense-mediated decay of mutant mRNA or mutant FOXC1 protein produced with 181 amino acids rather than full-length 553 amino acids missing the forkhead domain, the most important function part of the protein (p.Gln106Argfs*75, Figure 1B,C). Analysis of the parents revealed that this mutation was inherited from the proband’s father, since the mother had a wild-type sequence. The two affected sisters of the proband had the same variant as their father and brother. When we tested 104 unaffected ethnically matched controls, none had the mutation. In addition, no sequence variant was found in the *PITX2* gene.

### Clinical characteristics

Clinical evaluation of the four patients showed typical clinical phenotypes of ARS, including uncontrolled intraocular pressure, glaucomatous visual field defect, iridocorneal adhesion on gonioscopy, hypoplasia and marked atrophy of the iris, flattening of the midface, and broad, flat noses. Interestingly, in these four patients, who had glaucoma in both eyes, the eye with more extensive areas of anterior insertion of the iris into the trabecular meshwork with prominent iris processes presented with higher IOP and was more resistant to medical treatment, to the point that two of the patients needed surgical management. In addition, of both eyes affected by ARS, the eye with higher IOP presented a thicker central cornea with broad Haab’s striae and lower endothelial cell counts. These asymmetric ocular manifestations were identified in the early stages and the later stages of the disease in this family. None of the four patients had any abnormalities on dental panoramic radiograph, auditory function test, trans-thoracic echocardiography, or computed tomography of the head.

### Patient details

#### Patient I:1

**Figure 3:**
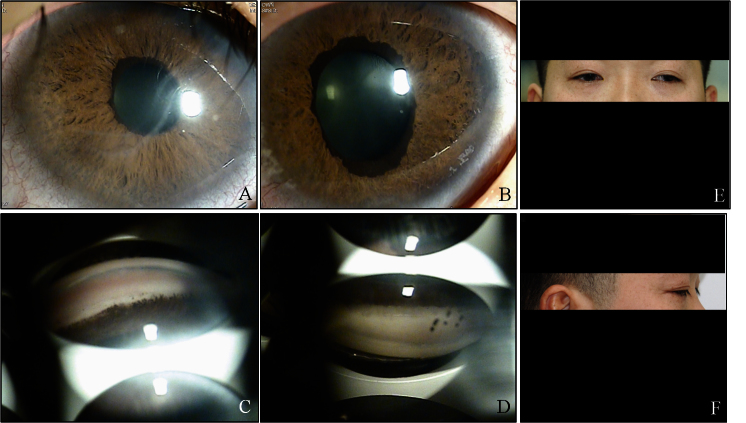
Ocular characteristics and systemic anomalies of patient I:1. Slit lamp photographs showed slight corneal edema, Haab’s striae, iridocorneal adhesions, and atrophy of the iris with corectopia in both eyes (**A**, **B**). Gonioscopy showed open angles in both eyes with anterior insertion of the iris into trabecular meshwork, prominent iris processes, and broad-based synechiae at places in both eyes (**C**, **D**). Physical examination revealed exotropia in the left eye, a flat midface, hypertelorism, and telecanthus (**E**, **F**).

A 40-year-old man was referred to our clinic because of uncontrolled IOP in the right eye. He was a known patient with glaucoma and had been treated by topical antiglaucoma medications in both eyes. However, he had been completely blinded in his left eye because of glaucoma 10 years earlier. He was referred to our center for IOP uncontrolled by topical medications in the right eye for 1 year. IOP measured with Goldmann tonometry was 26 mmHg in the right eye and 36 mmHg in the left. His best-corrected vision was 20/80 in the right eye with mild myopia. His left eye was deviated outward. Slit lamp examination revealed slight corneal edema, Haab’s striae, iridocorneal adhesions, and atrophy of the iris with corectopia in both eyes. Further physical examination revealed a flat midface, hypertelorism, and telecanthus. Other detailed ocular and systemic findings for this patient are shown in Table 1 and Figure 3. He had normal intelligence. Based on these findings, he was diagnosed with ARS. The IOP in the right eye was not adequately controlled with medication alone, so we performed trabeculectomy with mitomycin C in this eye. Postoperatively, at the third month, the IOP was well controlled in the right eye.

#### Patient II:1

A 12-year-old boy was referred to our clinic because of uncontrolled IOP in the right eye. He was born at 38 weeks' gestation with a birthweight of 2,500 g. The pregnancy and delivery were uneventful. The corneal diameter was 12 mm in both eyes. His best-corrected vision was 20/200 in the right eye and 20/20 in the left eye. IOP measured with Goldmann tonometry was 36 mmHg in the right eye and 24 mmHg in the left. Haab’s striae and iris hypoplasia were seen in the anterior segment. Gonioscopy showed open angles in both eyes with anterior insertion of the iris into the trabecular meshwork, prominent iris processes, and broad-based synechiae in places. Optic disc examination revealed a cup-to-disc ratio (CDR) of 0.9 in the right eye and 0.6 in the left. Humphrey visual fields revealed severe glaucomatous defects in the right eye, but the left eye was normal. The patient had apparent hypertelorism, telecanthus, a flat face, and a flat, broad nasal bridge. Other detailed ocular and systemic findings for this patient are shown in Table 1 and Figure 2. Because of these findings, he was diagnosed with bilateral ARS. He was treated with topical antiglaucoma medications in both eyes. The IOP in both eyes was well controlled on medication alone.

#### Patient II:2

**Figure 4:**
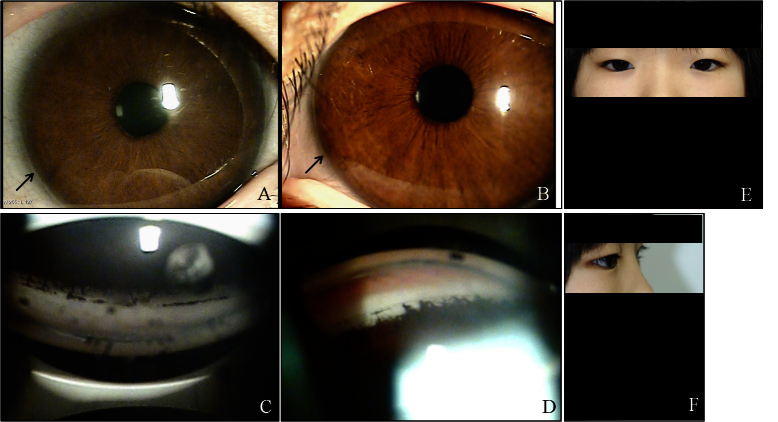
Ocular characteristics and systemic anomalies of patient II:2. Slit lamp photographs showed iris hypoplasia and posterior embryotoxon (black arrows) in both eyes, but Haab’s striae were observed only in the right eye (**A**, **B**). Gonioscopy showed open angles in both eyes with anterior insertion of the iris into the trabecular meshwork, prominent iris processes, and broad-based synechiae at places in both eyes (**C**, **D**). Physical examination revealed hypertelorism, telecanthus, a flat face, and a flat broad nasal bridge (**E**, **F**).

An 11-year-old girl was referred to our clinic because of uncontrolled IOP in the right eye. She was born at 37 weeks' gestation with a birthweight of 2,800 g. The pregnancy and delivery were uneventful. The corneal diameter was 11.8 mm in both eyes. Her best-corrected vision was 20/200 in the right eye and 20/20 in the left eye. IOP as measured with Goldmann tonometry was 32 mmHg in the right eye and 24 mmHg in the left. Iris hypoplasia and posterior embryotoxon were seen in both eyes, but Haab's striae were present only in the right eye. Gonioscopy showed open angles in both eyes, with anterior insertion of the iris into the trabecular meshwork, prominent iris processes, and broad-based synechiae in places. Optic disc examination revealed a CDR of 0.5 in the right eye and 0.3 in the left. Humphrey visual fields revealed severe glaucomatous defects in the right eye, but the left eye was normal. She had apparent hypertelorism, telecanthus, a flat face, and a flat, broad nasal bridge. Her other detailed ocular and systemic findings are shown in Table 1 and Figure 4. Because of these findings, she was diagnosed as having bilateral ARS. The IOP was not adequately controlled with medication alone in the right eye, so we performed trabeculectomy with mitomycin C in this eye. Postoperatively, at the fourth month, the IOP was well controlled in the right eye.

#### Patient II:3

**Figure 5:**
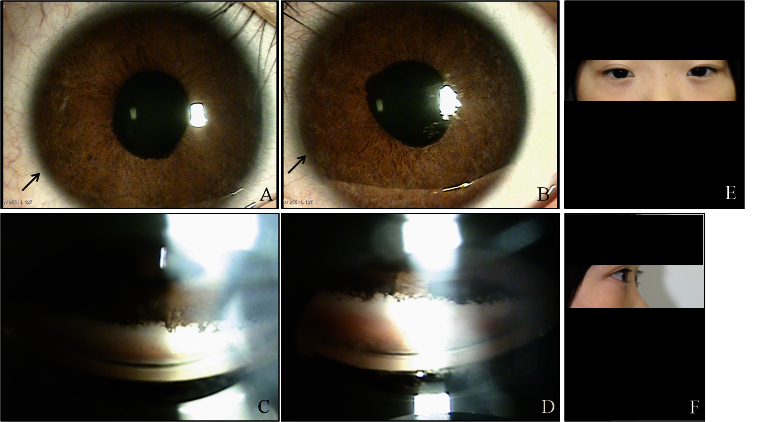
Ocular characteristics and systemic anomalies of patient II:3. Slit lamp photographs showed hypoplasia and iris posterior embryotoxon (black arrows) in both eyes, but Haab’s striae were observed only in the left eye. **A**, **B**: Gonioscopy showed open angles in both eyes with anterior insertion of the iris into the trabecular meshwork, prominent iris processes, and broad-based synechiae at places in both eyes (**C**, **D**). Physical examination revealed hypertelorism, telecanthus, a flat face, and a flat broad nasal bridge (**E**, **F**).

A 10-year-old girl was referred to our clinic because of uncontrolled IOP in the left eye. She was born at 38 weeks' gestation with a birthweight of 3,100 g. The pregnancy and delivery were uneventful. The corneal diameter was 11.5 mm in both eyes. Her best-corrected vision was 20/20 in both eyes. IOP, as measured with Goldmann tonometry, was 24 mmHg in the right eye and 30 mmHg in the left. Iris hypoplasia and posterior embryotoxon were seen in both eyes, but Haab's striae were seen only in the left eye. Gonioscopy showed open angles in both eyes with anterior insertion of the iris into the trabecular meshwork, prominent iris processes, and broad-based synechiae. Fundus examination revealed a CDR of 0.5 in the right eye and 0.5 in the left. Humphrey visual fields revealed paracentral scotoma in the left eye and a normal visual field in the right eye. She had apparent hypertelorism, telecanthus, a flat face, and a flat, broad nasal bridge. Other detailed ocular and systemic findings for this patient are shown in Table 1 and Figure 5. Because of these findings, she was diagnosed as having bilateral ARS. She was treated with topical medication in both eyes, and the IOP in both eyes was well controlled on medication alone.

## Discussion

In this study, we described a novel *FOXC1* gene mutation (c.317delA) that is expected to result in ARS through haploinsufficiency due to nonsense-mediated decay of mutant mRNA or the mutant FOXC1 protein produced (Figure 1B,C). Unidentified until now, the mutation was found in a Korean family affected by autosomal dominant ARS. To the best of our knowledge, deletions of *FOXC1* have been described in 13 patients, but neither the c.317delA mutation nor the ocular and systemic abnormalities seen in the family comprising the present case series had yet been seen in patients with ARS [15]. These four patients demonstrated characteristic clinical phenotypes.

The conditions that comprise ARS are all inherited in an autosomal dominant pattern with high penetration. ARS is genetically heterogenous, and mutations in different genes lead to similar clinical phenotypes. ARS has been associated with mutations in two genes: *PITX2* at 4q25 and *FOXC1* at 6p25 [4,6,16]. A third locus was suggested by deletions in 13q14, supported by linkage analysis, but a disease-causing gene has not yet been identified [9,17]. In two isolated cases, deletion in the 16q23–q24 region and deletion of the *PAX6* gene at 11p13, respectively, were related to ARS, but these findings were not supported by other studies [18]. In more than 60% of patients, the genetic defect underlying ARS is not known [19].

Genetically, point mutations in the *FOXC1* gene cause ARS, and patients with the 6p25 deletion syndrome probably exhibit ARS because *FOXC1* is located on chromosome 6p25 [4,7,20]. The *FOXC1* gene is a member of the forkhead/winged helix family of transcription factors, which are characterized by an evolutionarily conserved DNA-binding domain and are essential for embryonic development [4,21]. The gene is expressed in human tissues, with the highest concentration in skeletal muscle, kidney, liver, and heart as well as the eyes [22]. In addition to the *FOXC1* gene, defects in other genes in the deleted region may also result in the ocular and non-ocular phenotypes of patients with the 6p25 deletion syndrome [23]. Gould et al. summarized the phenotypes of patients with 6p25 deletions, showing that these patients exhibited a recognizable pattern of malformations. These malformations include hypertelorism, downslanting palpebral fissures, ARS, hearing loss, anomalies in the central nervous system, and developmental delay [13]. Manifestations of the four patients described in this case series were consistent with those of the 6p25 deletion syndrome.

To our knowledge, intragenic mutations of *FOXC1* have been described in 46 patients, and these include missense (n=23) and nonsense (n=6) mutations, as well as deletions/insertions/duplications (n=17) [15]. These mutations in human *FOXC1* have been associated with anterior segment dysgenesis, iris anomalies, and developmental glaucoma. Interestingly, the same *FOXC1* mutation may lead to different clinical manifestations. For example, the Leu86Phe mutation led to ocular changes in one patient, but the same mutation in another patient led to several systemic abnormalities, including obesity, short stature, myocardial infarction, and dental abnormality [20,24]. Functional studies of *FOXC1* missense mutations do not imply a strong correlation between the protein function and the phenotype. Strungaru et al. suggested that patients with *FOXC1* duplication have a more severe prognosis for glaucoma development compared with patients with intragenic *FOXC1* mutations [24]. Although *FOXC1* clearly plays an important role in eye development, the complexities of this association are made evident by the lack of any conclusive genotype-phenotype correlations [25]. A better understanding of the relationship between these genetic lesions and clinical manifestations in patients with ARS would lead to better clinical management and genetic evaluation, but this relationship is complex and difficult. Individuals with *FOXC1* mutations or deletions demonstrate a spectrum of phenotypic consequences, including the same mutated allele seen in affected and unaffected members of the same family [26,27]. It has therefore been suggested that either environmental factors and/or modifier genes interact with *FOXC1* in producing a disease phenotype, and this has been seen with other ocular developmental genes [28,29]. This report is especially worthy of notice in that it showed different stages of ARS in a family.

The major clinical concern in ARS is the risk of developing sight-threatening glaucoma, which is estimated to occur in 50% of patients with ARS [2,30]. Further, only 18% of patients with glaucoma and either *FOXC1* or *PITX2* genetic defects respond to medical or surgical treatment [24]. Although physical occlusion of the angle structure is not a prerequisite for elevated IOP and glaucoma in ARS, the severity of glaucoma correlates with the level of iris insertion into the angle [30]. In the four patients of this series, who had glaucoma in both eyes, the eye with more extensive areas of angle closure presented with higher IOP and was more resistant to medical treatment. In addition, as shown in Figure 1, Figure 2, Figure 3, and Figure 4, the eye with higher IOP presented a thicker central cornea with broad Haab’s striae. Further functional studies may clarify whether the novel mutation of *FOXC1* found in our patients is associated with an increase in the thickness of the cornea or the formation of Haab’s striae.

In summary, this study described a novel mutation (c.317delA) in the *FOXC1* gene of four members of a Korean family who were affected by autosomal dominant ARS. All four patients with this until-now unidentified mutation demonstrated typical clinical phenotypes of ARS, including uncontrolled intraocular pressure, glaucomatous visual field defects, iridocorneal adhesion on gonioscopy, hypoplasia and marked atrophy of the iris, flattening of the midface, and broad, flat noses. Additionally, of both eyes affected by ARS, the eye with higher IOP presented more extensive areas of areas of anterior insertion of the iris into the trabecular meshwork with prominent iris processes and a thicker central cornea with broad Haab’s striae. We therefore suggest that the c.317delA mutation in *FOXC1* is a cause of typical ARS. Our results may be useful for a better understanding of the spectrum of *FOXC1* mutations and the role of *FOXC1* in the development and progression of ARS. In addition, identifying the genetic causes underlying ARS cases allows for more precise genetic counseling and better prognosis. In addition, in the future, gene-specific or mutation-specific therapies may become available.
